# Special Issue “Protein Analysis by Mass Spectrometry”

**DOI:** 10.3390/molecules28062541

**Published:** 2023-03-10

**Authors:** Simone König

**Affiliations:** IZKF Core Unit Proteomics, Interdisciplinary Center for Clinical Research, University of Münster, Röntgenstr. 21, 48149 Münster, Germany; koenigs@uni-muenster.de

## Spectral Data Quality in Mass Spectrometry-Based Proteomics

When the *Molecules* Assistant Editors invited me as a Guest Editor for the Special Issue “Protein Analysis by Mass Spectrometry”, I hesitated for several months, not only because of a busy schedule, but also because of the abundance of the literature on the topic. In each year, 2021 and 2022, SciFinder (a literature search software) contained ~56.000 papers on this topic in the English language alone; in 2022, it was ~10.000 articles for the search term “proteomics”. Not only the number of individual contributions in this area, but also the number of Special Issues with the same or similar topics are on the rise. In such a situation, the attempt to assemble a “special” collection of papers appears to be futile. I eventually accepted the challenge, because I figured that I could use the opportunity and spread the concerns about data quality in proteomics. As several authors including myself have discussed in the past [1,2,3], mass spectrometry has gained an important role not only in life science research, but also as evidence-generating analytical method in areas such as sports or forensic medicine. Improperly validated results, for instance, in tox-screens, can have serious consequences in courts of law [1]. In proteomics, as discussed by Coorssen and Yergey [2], the automation of both measurement and data analysis procedures, while being a great step forward in technology development, has led to an undue faith in software output followed by over-interpretation of results. The principles of analytical chemistry with respect to factors such as reproducibility, sensitivity and specificity, however, still apply. It is necessary for anybody working in the area of shot-gun protein analysis, be it a bioinformatician or a clinician, to acquire a basic knowledge about the composition and the quality of peptide fragment ion spectra in order to understand when the collected data are not suitable for further evaluation [3].

For this Issue, authors were asked to scrutinize their data in that respect and phrase their results more conservatively whenever evidence was weak. Besides classical proteomics works (on central retinal vein occlusion, severe acute pancreatitis, gastric cancer, COVID-19), the Issue contains methodological advances (N-glycopeptide analysis, urinary tract pathogen identification) and results from de novo peptide analyses (insect hormones, snake venom protease). These papers represent a tiny glimpse of current protein research in its different forms and showcase the impact of mass spectrometry.

## Data Availability

Not applicable.
